# Supplementary material for: Gentamicin induction of the gonococcal hicAB toxin-antitoxin-encoding system and impact on gene expression influencing biofilm formation and in vivo fitness in a strain-specific manner
Source: mBio. 2025 Jul 30;16(9):e01595-25. doi: 10.1128/mbio.01595-25 (PMC12421842; doi:10.1128/mbio.01595-25)
Supplement: Supplemental Material — Figures S1 to S5; Tables S1 to S5. [file mbio.01595-25-s0001.pdf]

Supporting Information

Contents

S1 Fig. Incubation with sub-lethal gentamicin reduces biofilm formation in from *N. gonorrhoeae.*

S2 Fig. The *hicAB* locus and gene expression in the presence of sub-lethal Gentamicin

S3 Fig. Protein alignment of HicA and HicB in multiple bacterial species

S4 Fig. Predicted Structure of the gonococcal HicA and HicB proteins

S5 Fig. The *hicAB* mutant shows reduced biofilm formation immediately after inoculation

S1 Table. Comparison of the gentamicin induced regulon to other published gonococcal regulons

S2 Table. MIC of *hicAB* mutant strains in FA19 and F62 backgrounds.

S3 Table. Bacterial strains and plasmids used in this study.

S4 Table. Primers used in this study.

S5 Table. Raw read statistics determined for gentamicin-untreated and -treated replicates

Supplemental References

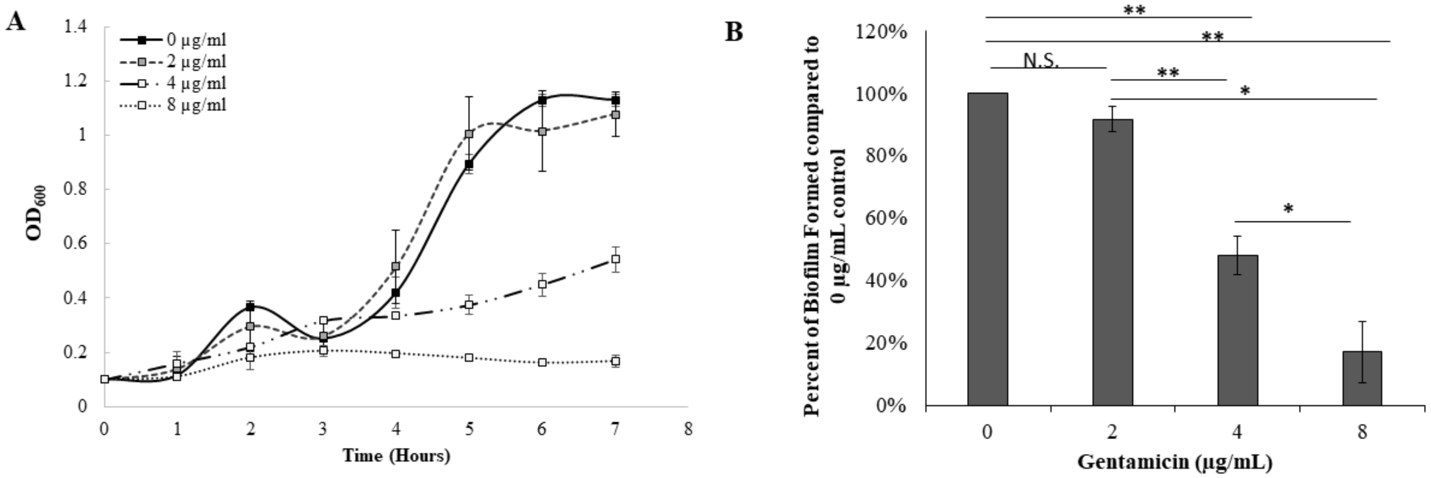

Fig S1. Incubation with sub-lethal gentamicin reduces biofilm formation in from *N. gonorrhoeae.* A) Growth of FA19 in the presence of increasing concentrations of Gen. B) 10^8 CFU/ml FA19 was incubated in 96-well tissue culture treated plates in the presence of increasing concentrations of Gen. Relative biofilm formation of the treated wells compared to the untreated 0 µg/mL control is shown as a percentage. Data calculated from quadruplicate wells from four independent experiments. Error bars show standard error. *, $P \leq 0.05$;**, $P \leq 0.01$; N.S., not significant.

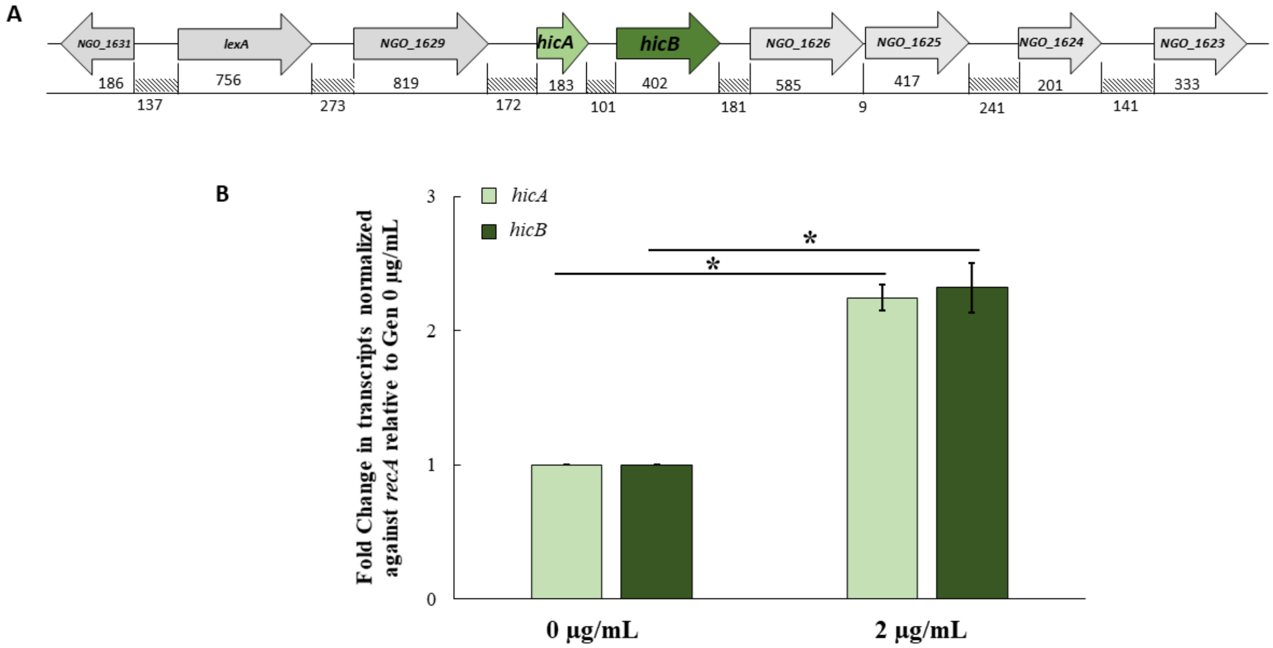

Fig S2. The *hicAB* locus and gene expression in the presence of sub-lethal Gentamicin. A) Genomic organization of the *hicAB* locus and its surrounding genes. *hicA* and *hicB* are highlighted in green. Black lines indicate start and stop codons. Gray spaces indicate intergenic regions. Numbers represent size in bp. Scale is approximate. B) Exposure to increasing concentrations of gentamicin increase *hicA* and *hicB* transcript levels. qRT-PCR analysis of RNA isolated after 4 hours of incubation with Gen from 10^8 CFU/ml of FA19 incubated in the presence of Gen at 2 µg/m. *, $P \leq 0.05$.; Data calculated from quadruplicate wells from four independent experiments. Error bars show standard error.

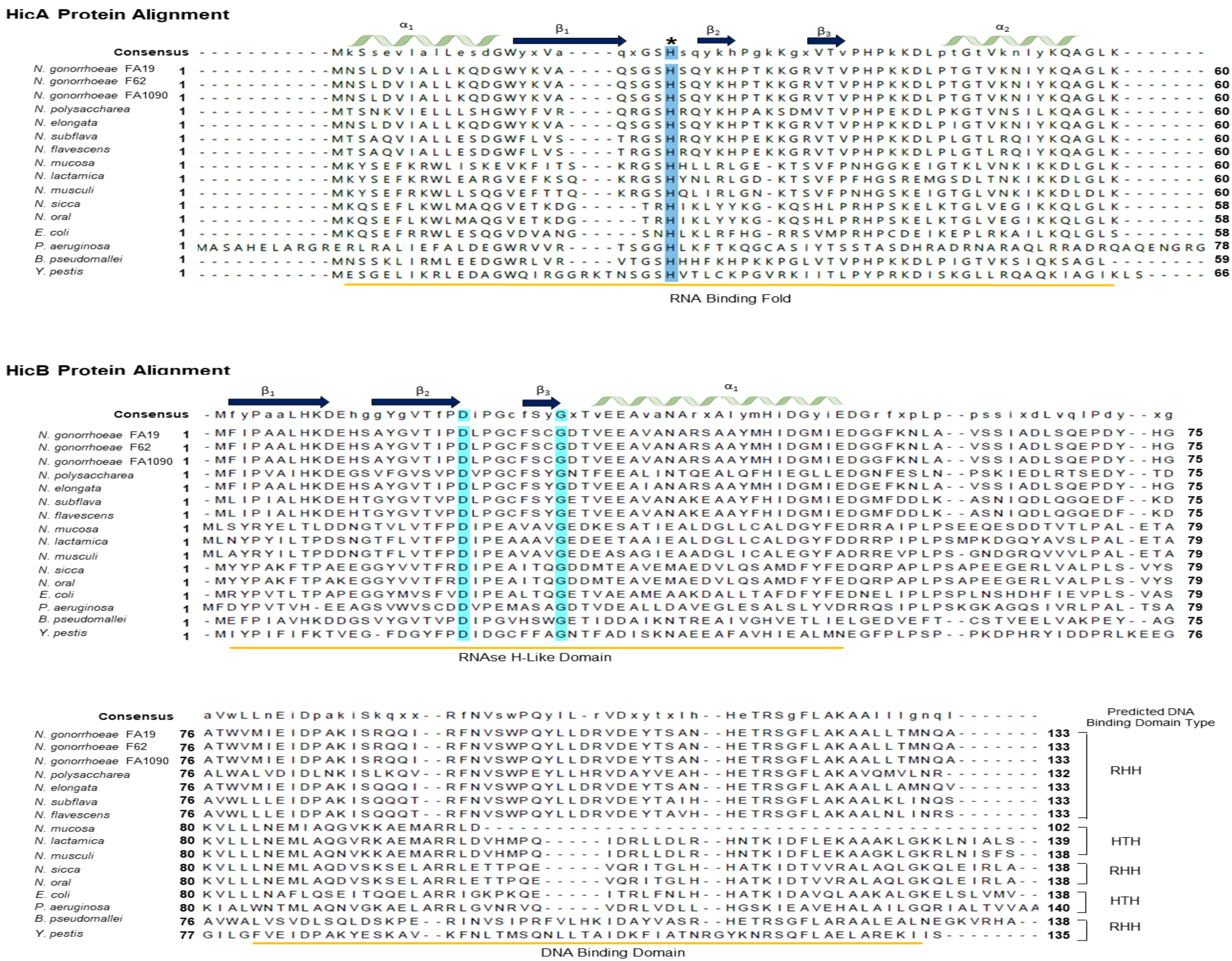
HicA Protein Alignment
Consensus
N. gonorrhoeae FA19
N. gonorrhoeae F62
N. gonorrhoeae FA1090
N. polysaccharea
N. elongata
N. subflava
N. flavescens
N. mucosa
N. lactamica
N. musculi
N. sicca
N. oral
E. coli
P. aeruginosa
B. pseudomallei
Y. pestis
RNA Binding Fold
HicB Protein Alignment
RNAse H-Like Domain
Predicted DNA Binding Domain Type
RHH
HTH
RHH
HTH
RHH
DNA Binding Domain

S3 Fig. Protein alignment of HicA and HicB in multiple bacterial species. Clustal W multiple sequence alignment of the HicA (upper panel) and HicB (lower panels) proteins in *N. gonorrhoeae* (FA19, F62 and FA1090), *Neisseria* commensals, *E. coli*, *P. aeruginosa*, *B. pseudomallei*, and *Y. pestis*. Consensus sequences are shown above each alignments. Secondary structures are shown above the alignment. Conserved residues are highlighted in blue. Domains are underlined in yellow. Predicted DNA binding domain subtype is indicated (RHH: Ribbon-Helix-Helix; HTH: Helix-Turn-Helix).

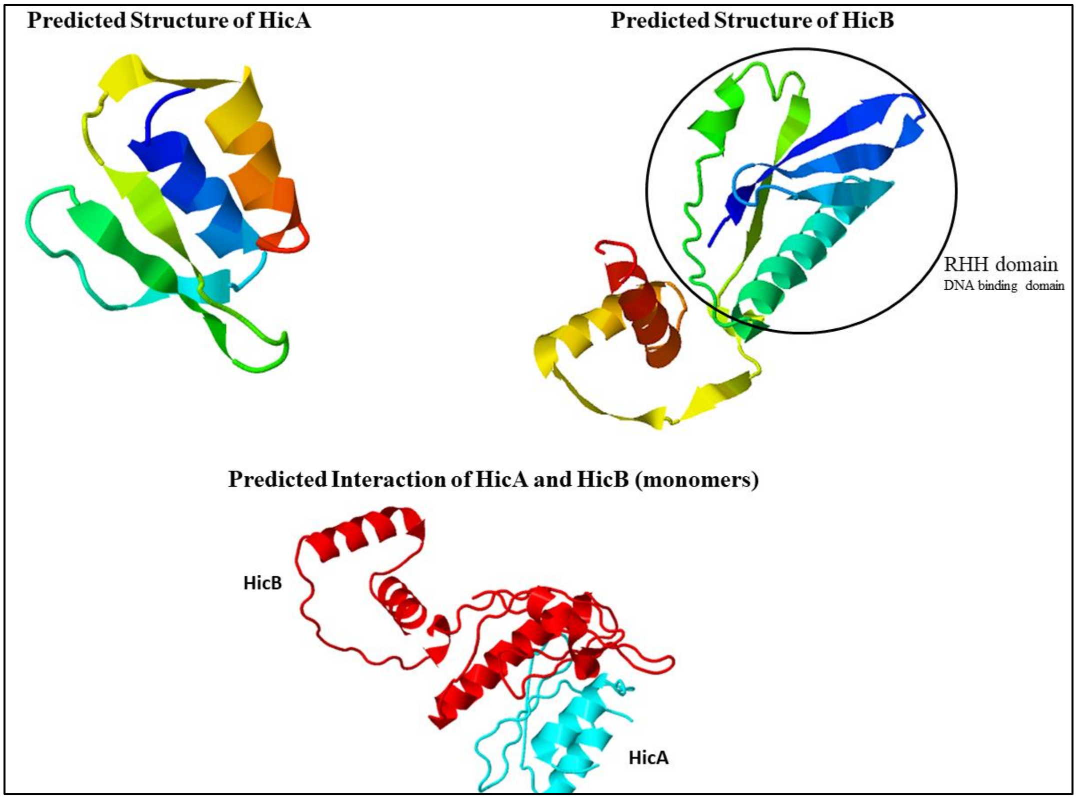

S4 Fig. Predicted Structure of the gonococcal HicA and HicB proteins. Three-dimensional ribbon structure of HicA and HicB from strain FA19. Protein was predicted by I-TASSER (https://zhanglab.ccmb.med.umich.edu/I-TASSER/) (1) using the predicted amino acid sequences. The Ribbon-Helix-Helix (RHH) DNA-binding domain was predicted by JPred4 (https://www.compbio.dundee.ac.uk/jpred4/index.html) (2) and is circled in black. Interaction between HicB and HicA monomers was performed using the PEPPI algorithm (https://zhanggroup.org/PEPPI/) (3).

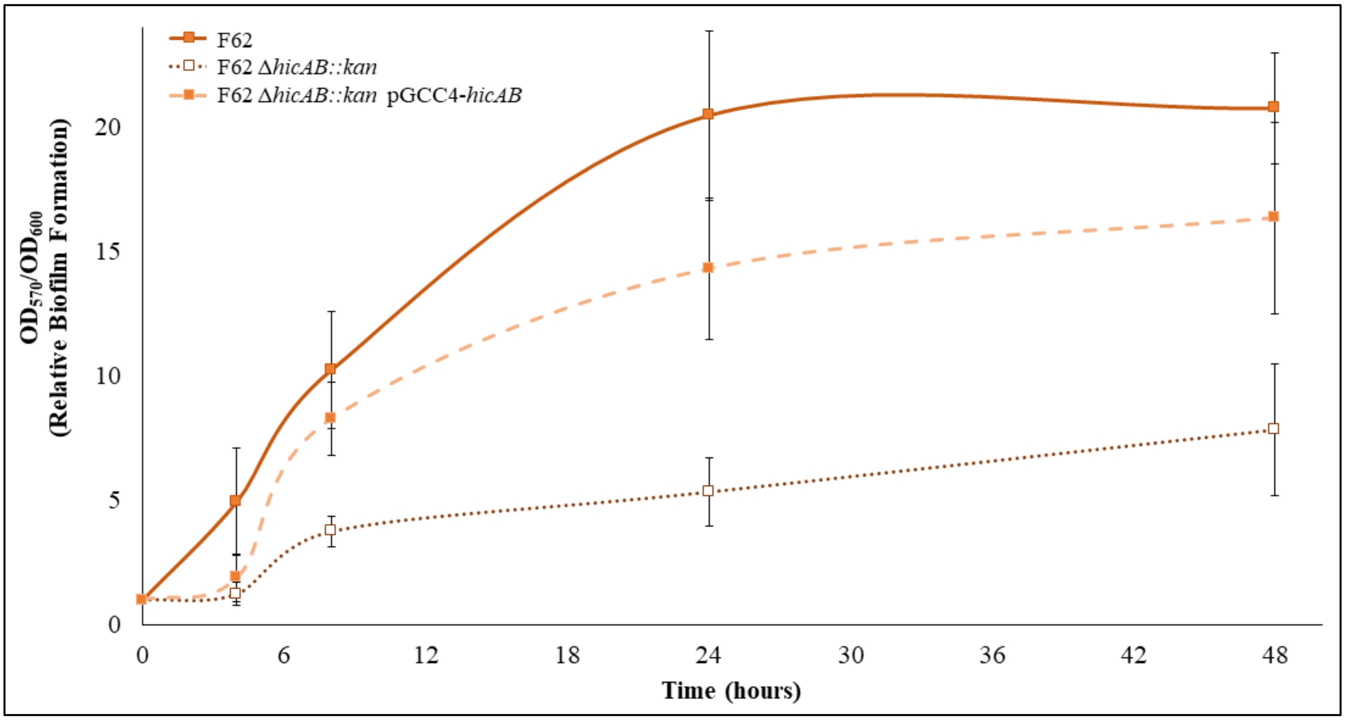

S5 Fig. The *hicAB* mutant shows reduced biofilm formation immediately after inoculation. Biofilm formation over a 48 hour time period. Comparison of growth between F62, F62 *hicAB::kan* and the complement strain. Relative biofilm formation is shown as a ratio of crystal violet stained biomass (OD_{570}) corrected by bacterial growth within in each well (OD_{600}). Data are representative of at least four independent experiments. Error bars show standard error.

S1 Table: Comparison of the Gentamicin Regulon to other Published Gonoccocal Regulons

Locus_tag	Product	Functional Classification	Regulons[a,b]										
			Heat Shock	RpoH	MpeR	H_2O_2	MtrR	NtrX	Anae-robic	LexA	NgoAx	MisR (FA1090)	MisR (FA19)
VT05_RS00725	protease HtpX	Heat Shock and Protein Folding	x						x			x	
VT05_RS01940	Fe-S cluster assembly transcriptional regulator IscR	Regulation						x					
VT05_RS02920	GNAT family N-acetyltransferase	Metabolism and Biosynthesis											
VT05_RS02925	DUF1778 domain-containing protein	Unknown											
VT05_RS03080	helix-turn-helix transcriptional regulator	Regulation							x		x		
VT05_RS04350	ATP-dependent chaperone ClpB	Heat Shock and Protein Folding	x	x	x	x	x		x		x		x
VT05_RS04935	tRNA-Ile	Metabolism and Biosynthesis											
VT05_RS05470	formylglycine-generating enzyme family protein	Unknown				x			x				
VT05_RS05625	tRNA-Ile	Metabolism and Biosynthesis										x	
VT05_RS06295	nucleotide exchange factor GrpE	Heat Shock and Protein Folding	x	x	x	x	x	x	x				x
VT05_RS06325	hypothetical protein	Unknown			x	x			x	x		x	
VT05_RS06330	molecular chaperone DnaK	Heat Shock and Protein Folding		x		x		x	x				x
VT05_RS06735	Pilin-like protein	Envelope Associated											
VT05_RS07005	carboxylating nicotinate-nucleotide diphosphorylase	Metabolism and Biosynthesis						x					
VT05_RS07010	hypothetical protein	Unknown				x			x				
VT05_RS07015	quinolinate synthase NadA	Metabolism and Biosynthesis											
VT05_RS07350	type II toxin-antitoxin system HicB family antitoxin	Transcription							x			x	

VT05_RS07355	type II toxin-antitoxin system HicA family toxin	Transcription							x			x	
VT05_RS07365	helix-turn-helix domain-containing protein	Regulation						x					
VT05_RS07890	tRNA-Ile	Metabolism and Biosynthesis											
VT05_RS09065	tRNA-Ile	Metabolism and Biosynthesis											
VT05_RS15295	IS5/IS1182 family transposase	Transcription											
VT05_RS15465	IS5/IS1182 family transposase	Transcription											

Comparison of genes differentially regulated in the presence of sub-lethal Gentamicin to genes found to be differentially regulated in other published RNA-seq or Microrray works.

[a]The Following Regulons were analyzed for overlap with the gentamicin regulon presented in this work: Heat Shock (4); RpoH (4); MpeR (5); H_2O_2 (6); MtrR (7); NtrX (8); Anaerobic (9); LexA (10); NgoAx (11); MisR –FA1090 (12), MisR-FA19 (13)

[b]Examined but no overlap found : Iron (14), NrrF (15), Fnr (16), MtrCDE (17)

S2 Table: MIC of *hicAB* mutant strains in FA19 and F62 backgrounds

	MIC (µg/mL)[ab]									
**Strain**	**GEN**	**PG**[c]	**CIP**	**RIF**	**CRO**	**AZI**	**FA**	**PMB**	**LL-37**	**H_2O_2**
**FA19**	8	0.008	0.064	0.125	<0.004	0.0625	0.0625	100	10	0.5
**FA19 *ΔhicAB::kan***	8	0.008	0.064	0.125	<0.004	0.0625	0.0625	100	10	0.5
**FA19 *ΔhicAB::kan* pGCC4-*hicAB***	8	ND	0.064	0.125	<0.004	2[d]	0.0625	100	10	0.5
**F62**	8	0.012	0.064	0.0625	0.0078	0.0625	0.125	200	12.5	1.0
**F62 *ΔhicAB::kan***	8	0.012	0.064	0.0625	0.0078	0.0625	0.125	200	12.5	1.0
**F62 *ΔhicAB::kan* pGCC4-*hicAB***	8	ND	0.064	0.0625	0.0078	2[d]	0.125	200	12.5	1.0

[a]Modal MIC values were determined from three of more independent assays unless otherwise noted; ND, not determined
[b]Abbreviations: Gentamicin (GEN); Benzylpenicllin (PG); Ciprofloxacin (CIP); Rifampicin (RIF); Ceftriaxone (CRO), Azithromycin (AZI), Fusidic Acid (FA), Polymixin B (PMB)
[c]Etest, n=1
[d]AZI resistance due to the ermC cassette in pGCC4 vector

S3 Table: Bacterial Strains and plasmids used in this study

Strain or plasmid	Genotype or description	Reference or source
N. gonorrhoeae		
FA19	WT strain	(18)
FA19 Δ*hicAB::kan*	FA19 containing the *hicAB* deletion	This Study
FA19 Δ*hicAB::kan* pGCC4-*hicAB*	FA19 Δ*hicAB::kan* expressing *hicAB* under control of an IPTG-inducible promoter at an alternate site in the chromosome	This Study
FA19 *rpsl*	WT strain with *rpsL* mutation encoding resistance to Str	(19)
FA19 *rpsl* Δ*hicAB::kan*	Transformant of FA19 *rpsl* strain containing the *hicAB* deletion	This Study
FA1090	WT strain	(20)
FA1090 Δ*hicAB::kan*	FA1090 strain containing the *hicAB* deletion	This Study
FA1090 Δ*hicAB::kan* pGCC4-*hicAB*	FA1090 Δ*hicAB::kan* expressing *hicAB* under control of an IPTG-inducible promoter at an alternate site in the chromosome	This Study
F62	WT strain	(21)
F62 Δ*hicAB::kan*	F62 strain containing the *hicAB* deletion	This Study
F62 Δ*hicAB::kan* pGCC4-*hicAB*	F62 Δ*hicAB::kan* expressing *hicAB* under control of an IPTG-inducible promoter at an alternate site in the chromosome	This Study
F62 *rpsl*	WT strain with *rpsL* mutation encoding resistance to Str	This Study
F62 *rpsl* Δ*hicAB::kan*	Transformant of F62 *rpsl* strain containing the *hicAB* deletion	This Study
Escherichia coli		
One Shot TOP10	F^- *mcr*A Δ(*mrr-hsdRMS-mcrBC*) ϕ80*lacZ*ΔM15 Δ*lacX74 recA1 araD139* (*ara leu*)*7697 galU galK rpsL* (Str^r) *endA1 nupG*	Invitrogen (Carlsbad, CA)
XL10-Gold Ultracompetent Cells	TetrΔ(*mcrA*)183 Δ(*mcrCB-hsdSMR-mrr*)*173 endA1 supE44 thi-1 recA1 gyrA96 relA1 lac* Hte [F´ *proAB lacIqZΔM15* Tn*10* (Tetr) Amy Camr]	Agilent Technologies (Santa Clara, CA)
BL21(DE3)pLysS	F^-*omp*T *hsd*S_B (r_B^-, m_B^-) *gal dcm* (DE3) pLysS(Cam^R)	Thermo Fisher Scientific (Waltham, MA)
**Plasmids**		
pBAD TOPO TA	Bacterial expression vector for one-step cloning of *Taq*-amplified PCR products	Thermo Fisher Scientific (Waltham, MA)

pBAD-*ΔhicAB*	pBAD containing the flanking region of the *hicAB gene* with a 671 bp deletion that leaves a scar region containing the *hicA* start codon and the *hicB* stop codon	This Study
pUC18K	pUC18 carrying the *apha3* non-polar cassette	(22)
pBad-*ΔhicAB::kan*	pBAD-*ΔhicAB* containing a *apha3* kan insertion in the scar region	This Study
pGCC4	IPTG-inducible Neisseria chromosomal complementation vector	(23)
pGCC4-*hicAB*	pGCC4 containing the wild type *hicAB* operon from FA1090, FA19 or F62	This Study
pET28a	Bacterial expression vector with T7lac promoter, N-terminal His-tag	Merck Millipore (Burlington, MA)
pET28a-HicB	pET-28a containing FA1090 *hicB* coding region	This Study
pET21a	Bacterial expression vector with T7lac promoter with N-terminal His-tag, N-terminal T7 tag and optional C-terminal His tag	Merck Millipore (Burlington, MA)
pET21a-HicA	pET-21a containing FA1090 *hicA* coding region	This Study

S4 Table: Primers used in this study

Primer Name	Sequence (5' to 3')	Purpose
recAqFw	AACCTCGAAGTCATTTCCACCGG	qRT-PCR
recAqRv	TCTGGCATTGGGCGACGGCTTC	qRT-PCR
grpE qRT_F	AAATGTGGAGGCGGTGGAAA	Validation of RNA-seq
grpE qRT_R	GCGCAGTTGTTCGTCTTTCA	Validation of RNA-seq
iscR qRT F	AAGTATGGGGGAGCTGTTG	Validation of RNA-seq
iscR qRT R	TTTTGAAACCGCTTGCCG	Validation of RNA-seq
htpX qRT F	TCGGCTTCACTGGTTCGATT	Validation of RNA-seq
htpX qRT R	TTGGGCTTCGACAGTGTTCA	Validation of RNA-seq
Ngo_0797_qRT F	CAAATTGACTCTGCCTGCC	Validation of RNA-seq
Ngo_0797_qRT R	CACATAAGTCCTGTCCAAACC	Validation of RNA-seq
fusA qRT F	CCGCTTGGCTAAAGAAGACC	Validation of RNA-seq
fusA qRT R	CAAGTGCAGCTCACCCATAC	Validation of RNA-seq
1982 qRTF	AAGATGTTAAAGCCATCCGTAAA	Validation of RNA-seq
1982 qRTR	ATTGAGTAACGCCGACCTT	Validation of RNA-seq
mtrE qRT F	TGTCTGCCTGCACCATGATT	Validation of RNA-seq
mtrE qRT_R	AGTGCGATGTCGATCAGCTT	Validation of RNA-seq
rpoH qRT F	AACGGCAGCCTCGAACAATA	Validation of RNA-seq
rpoH qRT R	GGTGGGACAGGATGAGTTGTT	Validation of RNA-seq
rmpM qRT F	AAGCCAAGGTCGCGTAGAAT	Validation of RNA-seq
rmpM qRT R	GGCGCGCAATGAATCCTTAT	Validation of RNA-seq
nadA qRT F	GAAAGACGAAATCAAGGT	Validation of RNA-seq
nadA qRT R	AAAGCCAAATCCTGAATC	Validation of RNA-seq
clpB qRT F	ACGATGCGAATGCCGAAG	Validation of RNA-seq
clpB qRT R	CCAATCAGCACGGGGTTG	Validation of RNA-seq
dnaK qRT F	ATGGCTCTGCAACGTCTGAA	Validation of RNA-seq
dnaK qRT R	CGAATTTGGCGCGGGTAATT	Validation of RNA-seq
hicA qRT F	AGCCTAGACGTTATTGCCC	qRT-PCR
hicA qRT R	GGTTACACGGCCTTTTTTTG	qRT-PCR
hicB qRT F	TAACCATTCCCGACCTTCC	qRT-PCR; Validation of RNA-seq
hicB qRT R	CCTCCGTCCTCAATCATACC	qRT-PCR; Validation of RNA-seq
HicABstop	TCTAGATAGCCAGCCCGTTCAACCC	Deletion of *hicAB;* generation of scar region
HicABRev	CACGTTGCCCATCCGTTCG	Amplification of *hicAB* operon
HicABFor	GGGCGTAACCCTCGCCC	Amplification of *hicAB* operon
HicABStart	TCTAGAAAGCTTCAATTATCACTCCTAATCT TAAT	Deletion of *hicAB;* generation of scar region
hicabpaci	TATTAATTAAAAATCATCAGAAACTTTGCC	Complementation of *hicAB;* cloning into pGCC4

hicabpmei	TAGTTTAAACAAATGGTATCGTTGTGC	Complementation of *hicAB;* cloning into pGCC4
aniA-q-Fw	GTGTCGTACTGCTCTTGATGGG	qRT-PCR
aniA-q-Rv	CAGCAATACTTGTACCACAAACAG	qRT-PCR
norB qRT F	CGAAAGCATCCTGCCTTACTATC	qRT-PCR
norB qRT R	GCGGGTGGTTTGCAACTT	qRT-PCR
nuoF qRT F	GCATTGCTTGAATCGTTGGAA	qRT-PCR
nuoF qRT R	GGGAACGGCGGTTTGAA	qRT-PCR
hicAB_FP_For	[FAM]-TCCCGACAAGGCACAAGAAA	DNAse I Protection Assay
hicAB_FP-Rev	[HEX]-CCCTTCCCTGCAACTACTGC	DNAse I Protection Assay
hicAB_P1_For	TAGGATCCGCTGACAGCCCTCAAGACC	*hicAB* EMSA probe
hicAB_P1_Rev	TAGGATCCTAGGCTATTCAATTATCACTCCTAAT	*hicAB* EMSA probe
rnpB1	ATACGGGGAGGAAAGTCCGGGCT	*rnpB* EMSA probe
rnpB2	TTCACCCTTGCCTGTGCTGCC	*rnpB* EMSA probe
norB EMSA F	TTCCTTTTGTAAGAAAAGTAGGG	*norB* EMSA probe
norB EMSA R	AGCTTCTTGTACTGTCCCAT	*norB* EMSA probe

S5 Table: Raw read statistics determined for Gentamicin-untreated and -treated replicates, for a total of 4 samples.

Sample Label	Sample #	Raw reads	Trimmed reads	Aligned reads to reference genome (%)	Reference genome[a]
FA19 - GEN	1	18,942,212	18,940,231	95.62	*N. gonorrhoeae* FA19
FA19 - GEN	2	18,567,273	18,478,999	98.33	*N. gonorrhoeae* FA19
FA19 + GEN	3	19,905,183	19,821,645	97.95	*N. gonorrhoeae* FA19
FA19 + GEN	4	20,140,688	20,041,713	96.49	*N. gonorrhoeae* FA19

[a]Reference genome: GenBank assembly accession: GCA_000273665.1

Supporting Information References

1. Zhou X, Zheng W, Li Y, Pearce R, Zhang C, Bell EW, Zhang G, Zhang Y. 2022. I-TASSER-MTD: a deep-learning-based platform for multi-domain protein structure and function prediction. Nat Protoc 17:2326-2353.
2. Drozdetskiy A, Cole C, Procter J, Barton GJ. 2015. JPred4: a protein secondary structure prediction server. Nucleic Acids Res 43:W389-94.
3. Bell EW, Schwartz JH, Freddolino PL, Zhang Y. 2022. PEPPI: Whole-proteome Protein-protein Interaction Prediction through Structure and Sequence Similarity, Functional Association, and Machine Learning. J Mol Biol 434:167530.
4. Gunesekere IC, Kahler CM, Powell DR, Snyder LA, Saunders NJ, Rood JI, Davies JK. 2006. Comparison of the RpoH-dependent regulon and general stress response in *Neisseria gonorrhoeae*. J Bacteriol 188:4769-76.
5. Hollander A, Mercante AD, Shafer WM, Cornelissen CN. 2011. The iron-repressed, AraC-like regulator MpeR activates expression of *fetA* in *Neisseria gonorrhoeae*. Infect Immun 79:4764-76.
6. Stohl EA, Criss AK, Seifert HS. 2005. The transcriptome response of *Neisseria gonorrhoeae* to hydrogen peroxide reveals genes with previously uncharacterized roles in oxidative damage protection. Mol Microbiol 58:520-32.
7. Folster JP, Shafer WM. 2005. Regulation of *mtrF* expression in *Neisseria gonorrhoeae* and its role in high-level antimicrobial resistance. J Bacteriol 187:3713-20.
8. Atack JM, Srikhanta YN, Djoko KY, Welch JP, Hasri NH, Steichen CT, Vanden Hoven RN, Grimmond SM, Othman DS, Kappler U, Apicella MA, Jennings MP, Edwards JL, McEwan AG. 2013. Characterization of an *ntrX* mutant of *Neisseria gonorrhoeae* reveals

a response regulator that controls expression of respiratory enzymes in oxidase-positive proteobacteria. J Bacteriol 195:2632-41.

9. Isabella VM, Clark VL. 2011. Deep sequencing-based analysis of the anaerobic stimulon in *Neisseria gonorrhoeae*. BMC Genomics 12:51.

10. Schook PO, Stohl EA, Criss AK, Seifert HS. 2011. The DNA-binding activity of the *Neisseria gonorrhoeae* LexA orthologue NG1427 is modulated by oxidation. Mol Microbiol 79:846-60.

11. Kwiatek A, Mrozek A, Bacal P, Piekarowicz A, Adamczyk-Poplawska M. 2015. Type III Methyltransferase M.NgoAX from *Neisseria gonorrhoeae* FA1090 Regulates Biofilm Formation and Interactions with Human Cells. Front Microbiol 6:1426.

12. Gangaiah D, Raterman EL, Wu H, Fortney KR, Gao H, Liu Y, Jerse AE, Spinola SM. 2017. Both MisR (CpxR) and MisS (CpxA) Are Required for Neisseria gonorrhoeae Infection in a Murine Model of Lower Genital Tract Infection. Infect Immun 85.

13. Kandler JL, Holley CL, Reimche JL, Dhulipala V, Balthazar JT, Muszynski A, Carlson RW, Shafer WM. 2016. The MisR Response Regulator Is Necessary for Intrinsic Cationic Antimicrobial Peptide and Aminoglycoside Resistance in *Neisseria gonorrhoeae*. Antimicrob Agents Chemother 60:4690-700.

14. Yu C, McClure R, Nudel K, Daou N, Genco CA. 2016. Characterization of the *Neisseria gonorrhoeae* Iron and Fur Regulatory Network. J Bacteriol 198:2180-91.

15. Jackson LA, Pan JC, Day MW, Dyer DW. 2013. Control of RNA stability by NrrF, an iron-regulated small RNA in *Neisseria gonorrhoeae*. J Bacteriol 195:5166-73.

16. Whitehead RN, Overton TW, Snyder LA, McGowan SJ, Smith H, Cole JA, Saunders NJ. 2007. The small FNR regulon of *Neisseria gonorrhoeae*: comparison with the larger

Escherichia coli FNR regulon and interaction with the NarQ-NarP regulon. BMC Genomics 8:35.

17. Ohneck EA, Goytia M, Rouquette-Loughlin CE, Joseph SJ, Read TD, Jerse AE, Shafer WM. 2015. Overproduction of the MtrCDE efflux pump in *Neisseria gonorrhoeae* produces unexpected changes in cellular transcription patterns. Antimicrob Agents Chemother 59:724-6.

18. Sarubbi FA, Jr., Blackman E, Sparling PF. 1974. Genetic mapping of linked antibiotic resistance loci in *Neisseria gonorrhoeae*. J Bacteriol 120:1284-92.

19. Jerse AE, Sharma ND, Simms AN, Crow ET, Snyder LA, Shafer WM. 2003. A gonococcal efflux pump system enhances bacterial survival in a female mouse model of genital tract infection. Infect Immun 71:5576-82.

20. Nachamkin I, Cannon JG, Mittler RS. 1981. Monoclonal antibodies against *Neisseria gonorrhoeae*: production of antibodies directed against a strain-specific cell surface antigen. Infect Immun 32:641-8.

21. La Scolea LJ, Jr., Dul MJ, Young FE. 1975. Stability of pathogenic colony types of *Neisseria gonorrhoeae* in liquid culture by using the parameters of colonial morphology and deoxyribonucleic acid transformation. J Clin Microbiol 1:165-70.

22. Menard R, Sansonetti PJ, Parsot C. 1993. Nonpolar mutagenesis of the *ipa* genes defines IpaB, IpaC, and IpaD as effectors of *Shigella flexneri* entry into epithelial cells. J Bacteriol 175:5899-906.

23. Mehr IJ, Seifert HS. 1998. Differential roles of homologous recombination pathways in *Neisseria gonorrhoeae* pilin antigenic variation, DNA transformation and DNA repair. Mol Microbiol 30:697-710.
